# Insight into the Phylogenetic Relationships of Phasmatodea and Selection Pressure Analysis of *Phraortes liaoningensis* Chen & He, 1991 (Phasmatodea: Lonchodidae) Using Mitogenomes

**DOI:** 10.3390/insects15110858

**Published:** 2024-11-03

**Authors:** Yuxin Chen, Yani Yuan, Wenhui Yang, Kenneth B. Storey, Jiayong Zhang, Danna Yu

**Affiliations:** 1 College of Life Science, Zhejiang Normal University, Jinhua 321004, China; 2 Department of Biology, Carleton University, Ottawa, ON K1S 5B6, Canada; 3 Key Lab of Wildlife Biotechnology, Conservation and Utilization of Zhejiang Province, Zhejiang Normal University, Jinhua 321004, China

**Keywords:** Phasmatodea, mitogenomes, phylogenetic relationship, origin time, selective stress analysis

## Abstract

Stick and leaf insects are a group among the Insecta that are famous for their extraordinary mimicry ability. Since the establishment of the Phasmatodea, their internal classification has been constantly revised. Mitochondrial genes as molecular markers have been widely used for species classification, but the phylogenetic relationships within the Phasmatodea remain to be thoroughly discussed. In the present study, five mitogenomes of Phasmatodea ranging from 15,746 bp to 16,747 bp in length were sequenced. Bayesian inference (BI) and maximum likelihood (ML) analyses were carried out based on a 13 PCGs data matrix (nt123) and a combined matrix of 13 PCGs and two rRNA genes (nt123_rRNA). The present study supports the conclusion that Phylliidae was the basal group of Neophasmatodea and confirms the monophyly of Lonchodinae and Necrosciinae, but it shows that Lonchodidae was polyphyletic. A sister group of Bacillidae and Pseudophasmatidae was also recovered. The phylogenetic tree based on the nt_123 dataset showed higher node support values. The construction of a divergent time tree in this study supported the conclusion that extant Phasmatodea originated in the Jurassic (170 Mya) and most lineages diverged after the Cretaceous–Paleogene extinction event. To explore whether the mitochondrial genes of *Phraortes liaoningensis* collected from high latitudes where low temperatures occur for eight months of the year are under selection pressure, this study used the branch-site model and the branch model to analyze the selection pressure on the 13 mitochondria protein-coding genes (PCGs). We found that both *ND2* and *ND4L* of *Ph. liaoningensis* exhibited positive selection sites using the branch-site model. This study shows that a low-temperature environment causes mitochondrial genes to be selected to meet the energy requirements for survival.

## Simple Summary

The phylogenetic relationship and origin time of Phasmatodea have been discussed by many researchers. In this study, five mitogenomes of Phasmatodea were newly sequenced and the disputes about their phylogenetic relationships were explored. Additionally, five reliable fossil calibration points were used to explore the origin and diversification time of Phasmatodea. Stick and leaf insects are widely distributed in tropical and subtropical regions, with only a few species distributed at high latitudes. We collected a *Phraortes liaoningensis* (Phasmatodea: Lonchodidae) from Anshan City, Liaoning Province, China, to analyze whether its mitochondrial genes were under selective pressure to adapt to a low-temperature environment.

## 1. Introduction

In response to predator pressure, stick and leaf insects (order Phasmatodea) have evolved extraordinary camouflage abilities, becoming the mimic masters of the Insecta. Mimicking branches or leaves is a typical characteristic of stick and leaf insects, and some species even disguise themselves as mosses [1]. Behaviorally, the predation risk is mitigated through nocturnal activities and mimicking the motion of swaying tree leaves [2]. Morphological similarities and sexual dimorphism within the Phasmatodea make classification based on morphological characteristics challenging [3]. Consequently, establishing a robust and high-level phylogenetic tree is crucial for understanding the evolutionary patterns of Phasmatodea.

Compared to other orders of Polyneoptera, Phasmatodea exhibits a moderate level of species diversity, with over 3500 species discovered worldwide, involving 14 families and 531 genera [4]. However, the taxonomic status of Phasmatodea remains controversial (both intra-order and inter-order). Within the Polyneoptera, several orders have been proposed as sister groups to Phasmatodea [5,6,7]. Recent analyses indicate that Embioptera is most likely the sister group to Phasmatodea [8,9,10,11,12], whereas Grylloblattodea or Mantophasmatodea also show evidence of a close relationship with Phasmatodea [13,14,15]. Numerous morphological and molecular data support the monophyly of the Phasmatodea [8,16,17,18,19]. The basal phylogenetic positions of Timematidea and Aschiphasmatidae within Phasmatodea are well-established and frequently corroborated [20,21,22]. Timematodea is the sister group of Euphasmatodea (the Phasmatodea excluding Timematodea) and Aschiphasmatidae is the sister group of Neophasmatodea (the Euphasmatodea excluding Aschiphasmatidae), which was restored by most phylogenetic analyses [23,24,25,26]. Beyond these robustly supported basal branches, the relationships among major phasmatodean lineages remain largely unresolved. Heteropterygidae comprises three subfamilies, and their phylogenetic relationships also remain unresolved. Researchers have proposed three distinct perspectives. Using extensive data from Heteropterygidae, Bank et al. [27] deduced that Dataminae was sister to (Heteropteryginae + Obriminae). Furthermore, the relationships of ((Dataminae + Obriminae) + Heteropteryginae) or (Obriminae + (Dataminae + Heteropteryginae)) are both supported by numerous studies [20,23,25,28]. Lonchodidae is the most species-rich taxonomic group, accounting for approximately one-third of the total species diversity within the order Phasmatodea [4]. In recent years, new species within this family have also been reported [4]. However, only 21 mitogenomes of Lonchodidae have been published on NCBI. Given this species richness, molecular data for this subfamily are scarce. The phylogenetic relationship between Lonchodinae and Necrosciinae has been discussed by many researchers [25,29]. Whereas some studies have restored their sister-group relationship [25,29,30], multiple studies based on mitogenomes have not confirmed this [14,16,31,32].

Insect mitogenomes are composed of a double-stranded circular structure ranging from 15 to 18 kb that encode 37 genes and are characterized by high replication rates, rapid evolutionary speed, and a conserved structure, which makes them excellent molecular markers for analyzing deep phylogenetic relationships [33,34,35]. With the rapid advancement in sequencing technologies, insect mitogenomes have been extensively reported and have become increasingly popular in phylogenetic studies, resolving numerous taxonomic issues [36]. As of July 2024, 57 mitogenomes of the Phasmatodea have been published by the National Center for Biotechnology Information (blast.ncbi.nlm.nih.gov/Blast.cgi, accessed on 1 July 2024), covering nine families of species. Compared to the species richness of this order, the mitogenome database of this group still requires further enrichment.

According to the comprehensive phylogenetic analysis conducted by Misof et al. [10], insects originated during the early Ordovician period, approximately 479 million years ago (Mya). Fossils provide crucial information about ancient organisms and can help calibrate the nodes (branching points) of the phylogenetic tree [37]. These calibrated nodes serve as time references, allowing researchers to estimate when specific evolutionary events occurred. Owing to the scarcity of precise time estimates and the ambiguous taxonomic status of fossils, the origin and diversification timeline of Phasmatodea has remained an unresolved enigma to date. Based on the results of previous research, the origin time of the Phasmatodea is approximately between the Triassic and Cretaceous periods. Using two fossil calibrations, Buckley et al. [3] estimated the origin of Phasmatodea to be approximately 95 Mya during the Cretaceous period, with the diversification of Euphasmatodea occurring around 51.9 Mya. Similarly, using four fossil calibrations, Bradler et al. [38] proposed an origin of Phasmatodea at 104 Mya, and Euphasmatodea was suggested to have emerged around 80.3 Mya. In addition, based on transcriptome data, Simon et al. [23] employed five fossil calibrations with results that supported that extant Phasmatodea began to diverge approximately 121.8 Mya. During the Cretaceous, the emergence and reproduction of various flowering plants formed a wide variety of plant morphology and structure [39,40,41]. Increased plant diversity can provide new ecological niches and resources for Phasmatodea. Recent studies have generally leaned towards an earlier origin for Phasmatodea. Tihelka et al. [42] provided support for the split between Timema and Euphasmatodea during the Triassic period. Forni et al. [14] traced the origins of stick and leaf insects back to the middle Permian period, approximately 273.8 Mya. Additionally, in the divergent time tree constructed by Bank et al. [18], the origin of Phasmatodea occurred in the Jurassic period, around 178.56 Mya. In 2022, Ghirotto et al. [43] counted and sorted ninety-five Phasmatodean fossils and proposed that *Araripephasma reliquum* is the oldest known Euphasmatodea fossil, with an estimated age of 115 Mya. Considering this perspective, the origin and diversification of Euphasmatodea may have occurred earlier. To gain insights into the origin of the Phasmatodea, accurate fossil node calibrations and a robust phylogenetic tree are crucial in inferring the timing of evolutionary events.

Stick and leaf insects prefer to inhabit warm climates areas [1], but some species have adapted to survive in colder alpine environments. In New Zealand, at least four species of stick and leaf insects have been identified to inhabit high-altitude regions [44,45]. For example, *Peruphasma marmoratum* was found in the highest point of the Cordillera de Mérida at altitudes ranging up to 5008 m above sea level [46]. These insects can survive and reproduce in this extremely high-altitude environment, highlighting their ability to withstand colder temperatures and more challenging conditions than those in low-latitude areas. In China, stick and leaf insects primarily inhabit the Oriental realm [4], while only a few are found in the Palaearctic region. Temperature has a significant effect on the physiological activities of ectotherms. The metabolic cold adaptation (MCA) theory suggests that, at the same temperature, the metabolism of species from cold climates is higher than that of species from warm climates [45]. Although the theory needs to be further proved, it has been demonstrated that temperature has an important effect on the aerobic metabolism of organisms [47]. The thirteen proteins encoded by the mitochondrial gene are all involved in the composition of the complex on the electron transport chain and provide 95% of the energy for the body through oxidative phosphorylation (OXPHOS) [48,49,50]. Temperature affects the energy requirements of organisms, which is a potential selection mechanism for the adaptive evolution of mitochondrial genes [51]. Although mitogenomes were traditionally considered to be selectively neutral [52], numerous studies have demonstrated that mitogenomes can undergo positive selection in response to changing environments [53,54,55]. For example, mites (*Tetranychus truncates*) are distributed in both warm and cold regions, and their *CYTB* and *ATP6* genes have positive selection sites [56]. Mayflies from high-latitude areas, such as those found in Ottawa, Canada, experience low-temperature stress with the result that there were 26 positive selection sites found on their mitogenomes [57].

To investigate the phylogenetic relationships, origin, and diversification time of Phasmatodea, five new mitogenomes of Phasmatodea were sequenced in this study. Additionally, five fossils were reassessed based on Tihelka et al. [42] and Ghirotto et al. [43]. We collected *Phraortes liaoningensis* (Phasmatodea: Lonchodidae) from high-latitude areas (Anshan City, Liaoning Province, China) to investigate whether there are positively selected sites on the mitochondrial genes of this species, which is adapted to low-temperature environments.

## 2. Materials and Methods

### 2.1. Samplie Collection and DNA Extraction

Five species were collected between 2021 and 2023 and identified by Zhang JY (College of Life Sciences, Jinhua, China). Details of the five species are shown in Table 1. Each sample was soaked in ethyl alcohol absolute at −20 °C and stored in the Zhang Laboratory, College of Life Sciences, Zhejiang Normal University, Jinhua, China. Total DNA from the left foreleg muscle tissue was extracted using the Ezup Column Animal Genomic DNA Purification Kit (Sangon Biotech Company, Shanghai, China). The extraction process was carried out following the provided instructions in detail.

### 2.2. Mitogenome Sequencing and Assembly

Mitogenome sequencing of *Ph. liaoningensis* used Sanger sequencing technology. According to the length of the amplified fragment, we used long-fragment polymerase chain reaction (PCR) or short-fragment PCR, respectively. The reagents and reaction procedures required for PCR were the same as those described by Zhang et al. [58]. The successful amplification products were detected by 1% agarose gel electrophoresis. The PCR products were sent to Sangon Biotech Company (Shanghai, China) for bidirectional sequencing. Primer Premier 5.0 [59] was used to design specific primers to complete the intervals between the amplified fragments, the specific primers are shown in Table S1. SeqMan in the DNASTAR Package v.7.1 [60] was used to rectify and splice nucleotide sequences.

Total DNA of *Pseudophasma subapterum*, *Sipyloidea biplagiata*, *Micadina brevioperculina*, and *Acanthophasma brevicercum* was sequenced using next-generation sequencing by BGI Tech Inc. (Shenzhen, China). Genomic DNA was randomly cut into fragments of the same size with nearly 150 bp, and adaptors were added to both ends. The genomic DNA was sequenced using the Illumina HiSeq 2000 platform (BGI Tech Inc. Shenzhen, China) with 150 bp paired-end reads. The size of the raw data ranged between 4 and 7 GB, and its quality was assessed using FastQC. To ensure the accuracy of mitogenome assembly, three splicing methods (NOVOPlasty v.4.2 [61], GetOrganelle v.1.7.1 [62] and MitoZ v.3.6 [63]) were used with all Phasmatodea sequences published on the NCBI for reference. After obtaining the same sequences, we annotated the mitogenomes.

### 2.3. Mitogenome Annotation and Basic Structure Analysis

MITOS2 (http://mitos2.bioinf.uni-leipzig.de/index.py, accessed on 24 May 2024) [64] was used for tRNA gene localization with the “reference” set as RefSeq 63 Metazoa. Based on the results from tRNAScan-SE (https://lowelab.ucsc.edu/tRNAscan-SE/index.html, accessed on 24 May 2024) [65] and MITOS2, the secondary structure of tRNA was predicted, and the results were visualized using Forna (http://rna.tbi.univie.ac.at/forna, accessed on 24 May 2024). Based on the start codons (ATN, GTG, TTG) and stop codons (T, TA, TAA, TAG), and with reference to the published lengths of PCGs in the phasmatodean mitogenomes available in NCBI, the localization of the 13 PCGs was determined. Based on the invertebrate codon, MEGA 7.0 [66] was performed to test whether the 13 PCGs could be successfully translated. Using the ClustalW alignment in MEGA 7.0 [66], we identified the two ribosomal RNA genes (*16S rRNA* and *12S rRNA*) by comparing the sequences with homologous genes from other phasmatodean mitogenomes. CG View (http://cgview.ca/, accessed on 24 May 2024) was used to map the mitogenomes [67]. PhyloSuite v.1.2.2 [68] was used to analyze the base composition (including GC-skew and AT-skew), amino acid usage, and relative synonymous codon usage of each mitogenome.

### 2.4. Phylogenetic Analyses

Using Grylloblattodea and Mantophasmatodea as outgroups [69], we constructed a phylogenetic tree comprising 60 stick and leaf insect species (including the 5 newly sequenced mitogenomes from this study and 55 sequences downloaded from NCBI, Table S2) [4,14,16,19,22,24,31,32,69,70,71,72]. In this study, PhyloSuite v1.2.2 [68] was employed to extract 13 PCGs, *16S rRNA*, and *12S rRNA*. The extracted genes were individually aligned using MAFFT v.7.475 [73]. Subsequently, Gblocks 0.91b [74] was applied to remove poorly aligned regions and select conserved regions. Finally, the concatenated nucleotide dataset was generated by combining the extracted genes. DAMBE 7.3.11 was used to analyze codon saturation for the 13 PCGs, and a substitution saturation analysis was performed using the method of Xia et al. [75]. Three positions of 13 PCGs did not exit the substitution saturation. For this research, two nucleotide data matrices were generated: (1) nt123 comprising 10,485 sites and (2) nt123_rRNA with 11,785 sites. Based on Bayesian information criteria (BIC), PartitionFinder 2.2.1 [76] was employed to partition and select the optimal substitution model of the two data matrices, respectively (Tables S3 and S4). The Bayesian Inference (BI) tree was reconstructed using MrBayes 3.2 [77], with the Markov Chain Monte Carlo analysis method, using 5 million generations sampling 1000 times and discarding the first 25% as burn-in. For maximum likelihood (ML) analysis, IQ-TREE v.2.1.2 [78] was employed with 1000 bootstrap replicates. The resulting evolutionary trees were visualized using FigTree v.1.4.

### 2.5. Phasmatodea Divergence Time Analysis

We selected five fossils that can be classified into the existing groups of Phasmatodea and referred to the age estimation of these fossils from previous studies [43,79,80,81,82]. Details of fossil information are available in Table 2. To speculate on the origin and divergence time of the existing Phasmatodea groups, we used MCMCTree [83] in PAML v4.8 [84] and the topological structure of the BI tree constructed based on the nt123_rRNA data matrix for time estimation. The root time was set to 326 Mya, which is the maximum age estimation of *Juramantophasma sinica* [42,85]. The base substitution rate was calculated using the baseml subroutine, and branch lengths were estimated using the maximum likelihood method. Based on the GTR model and the approximate likelihood calculation, MCMCtree was employed to estimate the divergence time, running for 1.4 million iterations. The first 400,000 iterations were discarded as burn-in, and samples were collected every 10 generations until 100,000 samples were obtained. Tracer v1.7.1 [86] was used to analyze the mcmc.txt file containing the effective sample size (ESS) generated by MCMCTree to ensure the convergence of results. Convergence was achieved when the ESS value was greater than 200. The output results are imported into FigTree v.1.4 for visualization [87].

### 2.6. Positive Selection Analysis of Mitochondrial Genes

The *Ph. liaoningensis* living in Anshan City, Liaoning Province, China, collected in this study experienced an average annual maximum temperature of 14.4 °C and a minimum temperature of 5.3 °C [88]. For nearly five months, the lowest temperature was below 0 °C. *Ph. liaoningensis* was selected as the foreground branch, and the remaining stick and leaf insects were selected as the background branch to investigate whether the 13 PCGs of *Ph. liaoningensis* were positively selected under a low-temperature environment. Branch model and branch-site model analyses were performed separately using the “preset running mode” in EasyCodeML v.1.07 [89]. The branch model compared two model hypotheses (the one-ratio mode and the two-ratio mode) using likelihood ratio tests (LRTs) to detect the heterogeneity of *ω* between the foreground branch and the background branches [90,91]. The branch-site model compares Model A with Model A_null_ to detect positively selected sites on the branch. When the LRT indicates the presence of positive selection, the Bayes empirical Bayes (BEB) was used to calculate the posterior probabilities of these sites. Sites with BEB values greater than 0.95 indicate potential positively selected sites. The secondary structures of proteins were predicted by SWISS-MODEL [92].

## 3. Results

### 3.1. Composition Analysis of Mitogenomes

**Figure 1:**
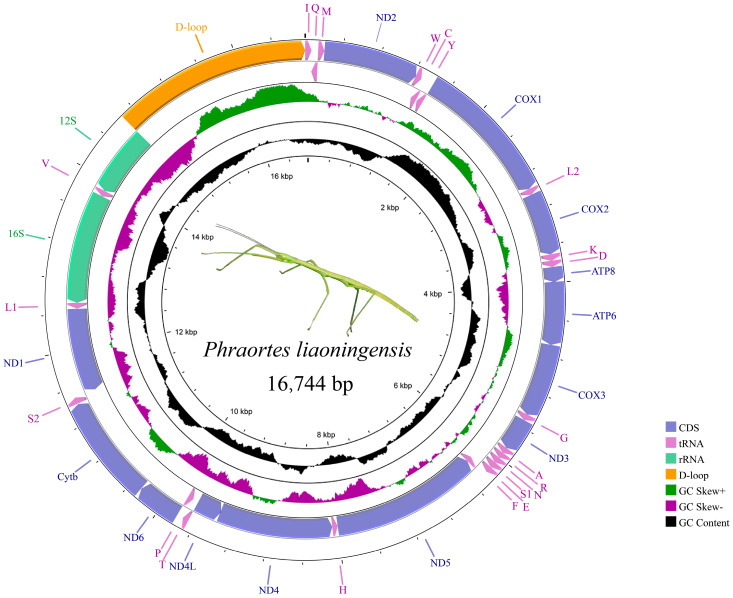
Mitogenome map of *Phraortes liaoningensis*. The outer circle genes are encoded by the J-stand and the inner circle genes are encoded by the N-strand. The 37 genes are represented by abbreviations, with tRNA genes represented by the corresponding amino acid abbreviations. GC content and GC-skew are plotted as the deviation from the average value of the entire sequence.

The present study obtained two complete mitogenomes and three mitogenomes lacking partial control regions, with lengths ranging from 15,746 bp to 16,744 bp. They all exhibited a structure consistent with ancestral insects, comprising 37 genes (13 PCGs, 22 tRNAs, and 2 rRNAs) and a control region, and taking *Ph. liaoningensis* as an example, the sequence of mitochondrial genes is shown in Figure 1. Except for eight tRNA genes, four PCGs, and two rRNA genes located on the negative strand, the remaining genes are all positioned on the positive strand. Overlaps and intergenic regions among genes range from 1 to 8 bp and 1 to 12 bp, respectively. There was a consistent overlap of 8 bp (AAGCCTTA) between *trnW* and *trnC* and an overlap of 4 bp (ATAA) between *ATP8* and *ATP6* (Table S5).

**Figure 2:**
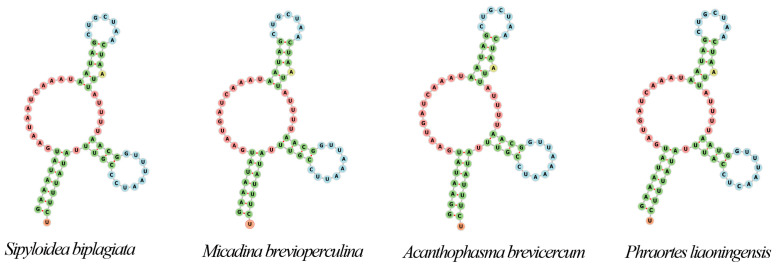
Secondary structure of trnS1 in *Sipyloidea biplagiata*, *Micadina brevioperculina*, *Acanthophasma brevicercum,* and *Ph. liaoningensis*. The DHU arm of the trnS1 secondary structure was lost in these species.

By analyzing the base composition of the five sequenced mitogenomes, we found that they exhibited a high AT content (over 70%). The PCGs located on the heavy strand showed a positive AT-skew and a negative GC-skew, whereas PCGs located on the light strand showed the opposite trend. Both *12S rRNA* and *16S rRNA* were located on the negative strand, with the length of *12S rRNA* ranging from 746 bp to 777 bp and the length of *16S rRNA* ranging from 1279 bp to 1290 bp. Both rRNAs showed a negative AT-skew and a positive GC-skew (Table S6). The PCGs of five newly sequenced sequences were analyzed. The *COX1* gene consistently had a length of 1534 bp across all sequences, with the lowest AT content ranging from 66.2% to 73.9%. Among all the PCGs, *ATP8* exhibited the highest AT content, ranging from 84.2% to 88.1% (Table S7). Almost all the PCGs were initiated by an ATN codon, but in some species, *COX1*, *ND1*, and *ND4* were initiated by the TTG start codon. TAA and the incomplete stop codon T are commonly used as termination codons (Table S3). Phe, Leu2, Ile, and Met are the amino acids most frequently encoded by the mitochondrial genes (Table S8). By analyzing the synonymous codon usage, it was found that the PCGs exhibited a preference for codons with U or A in the third position. UUU, UUA, AUU, and AUA are among the absence of a frequently utilized codon, each being used more than 250 times (Figure S1). Except for the DHU arm absence observed in *trnS1* of some species (*S. biplagiata*, *M. brevioperculina*, *A. brevicercum*, and *Ph. liaoningensis*), the tRNA secondary structures exhibit the typical cloverleaf structure (Figure 2).

### 3.2. Phylogeny Analyses of Phasmatodea

**Figure 3:**
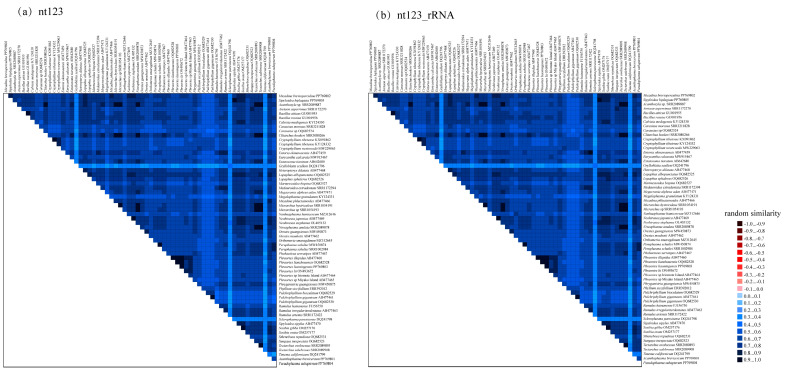
Sequence heterogeneity analyses of nt123 and nt123_rRNA datasets, color depth indicates the level of random similarity.

**Figure 4:**
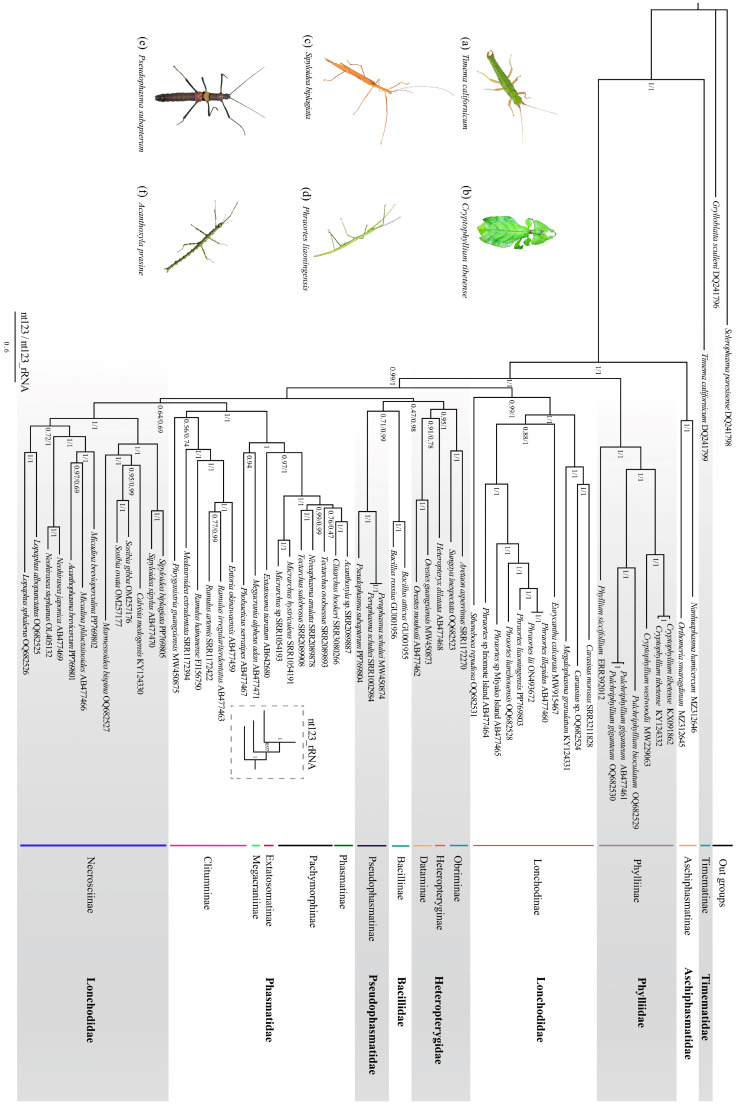
Phylogenetic relationships of Phasmatodea among 60 species were inferred from BI analyses based on nt123 and nt123_rRNA datasets, using *Sclerophasma paresisense* and *Grylloblatta sculleni* as outgroups. The topological structure differences between the BI trees constructed by the two datasets are shown in the dotted box. GenBank or sequence read archive (SRA) numbers were annotated after the species name. The node value on the branch represents the posterior probability, the left number is based on the analysis of the nt123 dataset, and the right number is based on the analysis of the nt123_rRNA dataset. The taxonomic ranks of the species are provided on the right side of the figure. The families and subfamilies to which the species pertain are provided on the right side of the figure.

**Figure 5:**
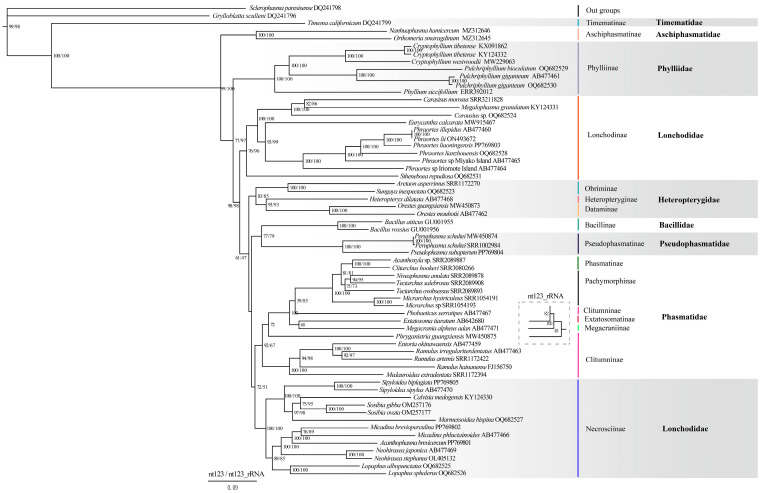
Phylogenetic relationships of Phasmatodea among 60 species were inferred from ML analyses based on nt123 and nt123_rRNA datasets, using *Sclerophasma paresisense* and *Grylloblatta sculleni* as outgroups. The topological structure differences between the BI trees constructed by the two datasets are shown in the dotted box. GenBank or SRA numbers were annotated after the species name. The node value on the branch represents the bootstrap value, the left number was based on the analysis of the nt123 dataset, and the right number was based on the analysis of the nt123_rRNA dataset. The taxonomic ranks of the species are provided on the right side of the figure. The families and subfamilies to which the species pertain are provided on the right side of the figure.

The sequence heterogeneity of the nt123 and nt123_rRNA datasets was analyzed by AliGROOVE v.1.07. The results showed that the random similarity value of the two datasets was positive (Figure 3), so it was suitable for the phylogenetic relationship analyses. The codon saturation of 13 PCGs was evaluated, and the results showed that three codons were unsaturated (Table S9). Using GTR + I + G as the best model, BI and ML trees were constructed based on two datasets (Figure 4 and Figure 5). These phylogenetic trees recovered nearly the same topology, and the difference was only in the phylogenetic status of several species (Figure 4 and Figure 5). Compared to the phylogenetic tree constructed using the nt123 dataset, the tree built using the nt123_rRNA dataset exhibited higher node confidence values. In both BI and ML analyses, the phylogenetic positions of Heteropterygidae, Bacillidae, and Pseudophasmatidae were different. The BI analysis supported a sister-group relationship between Heteropterygidae and (Bacillidae + Pseudophasmatidae). However, (Bacillidae + Pseudophasmatidae) clustered with (Phasmatidae + Lonchodidae) in the ML analysis. The phylogenetic trees consistently recovered Timematidae and Aschiphasmatidae in basal positions within the Phasmatodea phylogenetic relationships. In our analyses, the monophyly of Phylliidae was recovered and it was positioned as a basal lineage within Neophasmatodea. Lonchodidae consists of two subfamilies (Lonchodinae and Necrosciinae), but it did not form a sister-group relationship. Necrosciinae and (Phasmatinae + Pachymorphinae + Extatosomatinae + Megacraniinae + Clitumninae) were recovered as sister groups, though this relationship had relatively low node support. In our phylogenetic analyses, Phasmatidae was a monophyletic group, but most of its subfamilies were parapyhletic groups. Heteropterygidae was resolved as a monophyletic group and its three subfamilies recovered a topological structure of ((Heteropteryginae + Dataminae) + Obriminae). Bacillidae and Pseudophasmatidae were identified as sister groups, but this association lacked strong node support.

### 3.3. Divergence Times of Phasmatodean Lineages

**Figure 6:**
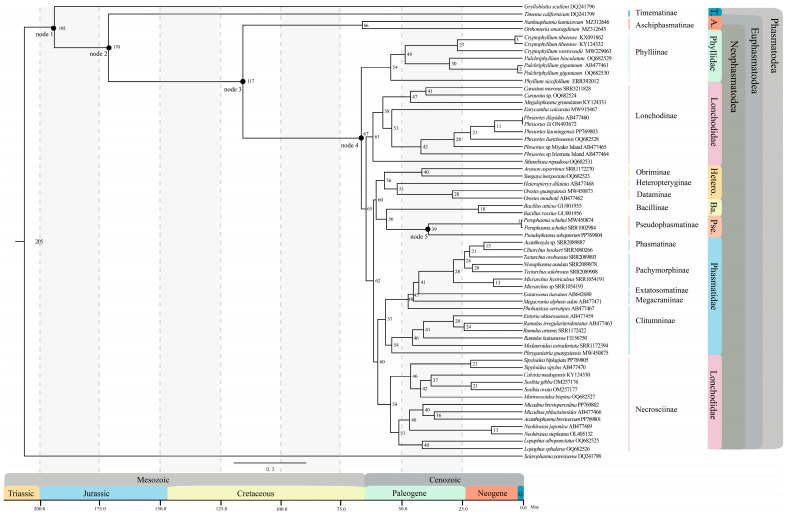
The divergence time tree for the Phasmatodea inferred from the nt123_rRNA dataset based on phylogenetic analyses using five fossil calibration points. The number on the node represents the divergence time. The geological time scale is shown below the figure and the unit is Mya. The position of fossil calibration points is represented by black dots within the divergent time tree. node1: *Adjacivena rasnitsyni*, node2: *Tumefactipes prolongates*, node3: *Araripephasma reliquum*, node4: *Eophyllium messelense*, node5: *Eophasmodes oregonense*. The full names of the abbreviations in the figure are as follows: Timematidae (T.); Aschiphasmatidae (A.); Heteropterygidae (Hetero.); Pseudophasmatidae (Pse.); Bacillidae (Ba.); Quaternary (Q.).

The BI tree constructed based on the nt123_rRNA dataset was used to estimate the origin and diversification timing of the Phasmatodea (Figure 6). Our study suggested that the extant Phasmatodea originated in the Jurassic around 170 Mya, which was also the time of divergence between Timematidae and Euphasmatodea. However, this node had a broad confidence interval (95% CI: 144–212 Mya). Aschiphasmatidae and Neophasmatodea diverged during the Cretaceous period at approximately 117 Mya (95% CI: 115–121 Mya). The Aschiphasmatidae later radiated around 66 Mya (95% CI: 44–101 Mya). At the Cretaceous–Palaeogene (K-Pg) boundary, around 67 Mya (95% CI: 64–70 Mya), Nophasmatodea started extensive diversification. Furthermore, the majority of the extant Phasmatodean families were established and experienced rapid radiation during the Paleogene. *Bacillus* was the most recently established lineage, originating around 18 Mya (95% CI: 10–29 Mya). Its sister clade Pseudophasmatidae exhibits a later origin, estimated at 39 Mya (95% CI: 31–45 Mya). The most recent common ancestor (MRCA) of Phylliidae was estimated at 54 Mya (95% CI: 47–61 Mya), coinciding with the origin time of Necrosciinae. Lonchodinae emerged around 54 Mya (95% CI: 49–59 Mya). The origin time of Heteropterygidae and Phasmatidae was similar, at 56 Mya and 57 Mya, respectively, both later experiencing rapid radiation.

### 3.4. Selective Pressure Analysis

**Figure 7:**
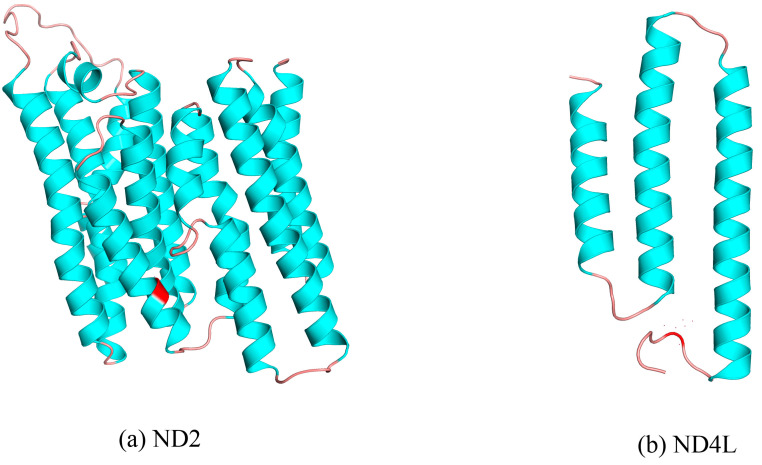
The protein secondary structure of *ND2* and *ND4L* from *Ph. liaoningensis* was predicted by SWISS-MODEL, and the corresponding selection sites are marked in red.

We conducted a selection pressure analysis on the mitochondrial 13PCGs, using *Ph. liaoningensis* as the foreground branch and the other Phasmatodea species as the background branches. The results of the branch model analysis indicate that in both model assumptions (two-ratio model and one-ratio model), the *ω* value was less than 1. Using LRT to compare the two models, a *p*-value of less than 0.01 indicated that the foreground branch has a significantly different *ω* value from the background branches, with a value of 0.02157 (Table S10). The analysis of the branch model results suggested that *Ph. liaoningensis*, as the foreground branch, was not subject to positive selection. In the analysis using the branch site model, LRT was employed to compare the two hypotheses: Model A and Model A _null_ (Table 3). With a *p*-value less than 0.05, the results lean towards Model A. The results indicated that the background branch *ω* value (*ω*0) was not greater than 1, implying no positive selection, whereas the foreground branch *ω* value (*ω*1) was greater than 1, suggesting positive selection. In the branch model, positive selection was not detected in the branch of *Ph. liaoningensis*. Two positive selection sites were detected in *Ph. liaoningensis* using the branch-site model. Among the 3653 amino acid sites, the BEB method identified two positively selected amino acid sites in the foreground branch. The 2483th and the 2107th amino acid positions, with a posterior probability of 0.996 and 0.967 (exceeding the standard threshold of 0.95). These two positive selection sites correspond to the 149th amino acid site of the *ND2* gene and the 84th amino acid site of the *ND4L* gene of *Ph. liaoningensis* (Figure 7). In the positive selection site of the *ND2* gene, the residue of this site in the foreground branch (*Ph. liaoningensis*) was aspartic acid (A), whereas the residue of this site in the background branch was various. In the positive selection site of the *ND2* gene, this site encodes valine in *Ph. liaoningensis* and was located on the α-helix. In the positive selection site of the *ND4L* gene, the residue of this site in the background branch was aspartic acid (D), whereas the residue of this site in the foreground branch was lysine (L).

## 4. Discussion

### 4.1. Phylogenetics of Phasmatodea

Based on the nt123 and nt123_rRNA datasets, BI and ML trees were constructed to analyze the relationships between different taxa within the Phasmatodea. In our analysis, Timematidae and Aschiphasmatidae were strongly supported as the basal status in the phylogenetic tree of Phasmatodea. Timematidae, a small wingless insect, was morphologically distinct from other stick and leaf insects by having three tarsal segments [81]. Molecular phylogenetic studies consistently confirm the sister-group relationship between Timematodea and Euphasmatodea, making Timematodea a stable taxonomic group in the Phasmatodea [18,23,42]. Necrosciinae was the most abundant subfamily, with about 840 described species in 113 genera [4], and the taxonomic relationship between Necrosciinae and Lonchodinae had been explored. Lonchodinae and Necrosciinae were originally classified as related taxa in the Heteronemiidae [93]. In 2008, Hennemann and Conle transferred Lonchodinae into the Phasmatidae [94]. In 2014, Bradler et al. revisited the classification of genera in two subfamilies, formally transferring *Neohirasea* into Necrosciinae and *Baculofractum* into Lonchodinae, and supported the conclusion that *Leprocaulinus* belongs to Lonchodinae [29]. In 2018, Robertson et al. formally classified Necrosciinae and Lonchodinae into the Lonchodidae [25]. The morphology of these two subfamilies has been frequently discussed. Sellick et al. suggested that Necrosciinae was polyphyletic, due to the heterogeneity of its egg-capsule morphology and egg-laying strategies [95]. However, many studies based on molecular data support the monophyly of these two subfamilies [3,25,29,38]. In our research, the monophyly of Lonchodinae and Necrosciinae was confirmed. However, their sister group relationship remained unconfirmed, and these two subfamilies have no stable phylogenetic position [14,72,96]. In 2022, Bank and Bradler [18] recovered the sister relationship between Lonchodinae and Necrosciinae using both partial mitochondrial and nuclear genes. Tihelka et al. [42] also recovered this relationship using transcriptome data. The sister group relationship between Lonchodinae and Phylliinae has also been restored in many studies based on mitogenomics [14,16,31,96], although morphological similarities between the two subfamilies have not been found. In our study, Phylliinae is located at the base of Neophasmatodea, as seen in previous analyses [26,27,38,70]. Except for Phylliinae, the remaining species in Neophasmatodea are the sister group to Lonchodinae. Some species of Lonchodidae (primarily from the Necrosciinae) clustered as sister groups with Phasmatidae which is consistent with a few other studies [97]. The sister group relationship between Lonchodinae and Necrosciinae was supported by numerous large-scale phylogenetic trees constructed based on data from seven molecular markers (*18S RNA*, *28S RNA*, *12S RNA*, *16S RNA*, *H3*, *COI*, and *COII*) [25,29,30]. Pseudophasmatidae and Bacillidae clustered into one clade, which was recovered in the phylogenetic trees constructed from both datasets in this study, but this conclusion has not been observed in other studies. Recent molecular studies have discovered that Pseudophasmatidae and Agathemeridae form a sister group. These two families and the Diapheromeridae are located at the base of the Neophasmatodea [23,98]. However, samples of Agathemeridae and Diapheromeridae were not collected in this study, so they could not be included in the analysis. The monophyly of Heteropterygidae and the phylogenetic topology of (Obriminae + (Heteropteryginae + Dataminae)) were recovered in our study, which was consistent with previous studies [14,20,23]. Comparing phylogenetic trees constructed from nt123 and nt123_rRNA datasets, revealed nearly identical phylogenetic topologies. This was interesting because rRNA genes have different evolutionary rates between species and have been widely used in phylogenetic relationship analysis of species [99,100,101]. The phylogenetic tree constructed from the dataset that included rRNA genes in our study had higher node support values. To clarify relationships within Phasmatodea, choosing a reliable dataset and incorporating more mitochondrial genes from diverse species is essential.

### 4.2. The Origin and Diversification of the Phasmatodea

Fossils with clear classification and age estimation are crucial for accurately estimating the origin and diversification of stick and leaf insects. The majority of fossils of Phasmatodea have been discovered from the Early Cretaceous to the Paleogene. *Eophyllium messelense* is the only undisputed oldest Phylliidae fossil to date and is often used to estimate the timing of differentiation within Phasmatodea [82]. The complete fossil record of Phasmatodea is relatively scarce and most fossils only record wing morphology, which makes accurate classification difficult and also brings challenges to estimating the origin and divergence time of Phasmatodea. To provide an evolutionary time framework of Phasmatodea, we combined fossil calibration points used in recent studies and added the *Araripephasma reliquum* comb. nov revised by Ghirotooa as a stem node for Euphasmatodea [43]. We constructed a divergence time tree, inferring the origin of the Phasmatodea in the Jurassic (170 Mya) and the origin of Euphasmatodea in the Cretaceous (117 Mya). This result is close to the time estimated by Bank et al., who supported the claim that Phasmatodea originated 178.56 Mya and that Euphasmatodea diversification began in the Cretaceous (106.13 Mya) [18]. Timematodea was the first to diverge from the remaining Phasmatodea and was the most ancient lineage within the Phasmatodea. This conclusion has been confirmed by many studies [23,25,38,42]. Previous research generally supported an earlier origin of the Phasmatodea, between the Cretaceous and Paleogene, and diversification occurring during the Paleogene [3,23,38]. Recent studies, including those utilizing transcriptomic data estimates, suggest that Phasmatodea originated between the Permian and Triassic periods, with significant diversification occurring during the Cretaceous [14,18,42,102].

Stick and leaf insects are herbivorous insects with an astonishing capability to mimic plants, and their origin and evolution are closely related to plant life. Fossil evidence dating as early as the Cretaceous shows the imitation of gymnosperm leaves by stick and leaf insects [103]. Modern Phasmatodea has an inseparable relationship with angiosperms; this is especially the case for the Phylliidae, which possess unparalleled leaf-mimicking abilities [26]. The mimicry of angiosperm leaves was a result of the more recent diversification of flowering plants. Fossil evidence indicates that the imitation of angiosperm leaves appeared in the Eocene, 47 Mya [82]. The original timing of angiosperms remains contentious. Based on fossil and molecular data, the origin of crown angiosperms were dated between 140 and 270 Mya [104]. The earliest angiosperm fossils trace back to the early Cretaceous [105]. After the K-Pg extinction event (65.5 Mya), global climate and vegetation types have undergone dramatic changes [106], and angiosperms have become increasingly dominant in terrestrial ecosystems, providing many new ecological niches for animal diversification [107]. In this study, most of the phasmatodean lineages were established after the K-Pg extinction event. This suggests that early diverging lineages might have been lost during the K-Pg extinction event and the rich variety of angiosperms provides the basis for the diversification of Phasmatodea.

### 4.3. Selection Pressure Analysis Based on Mitochondrial Genes

Temperature exerts a significant influence on the survival of ectotherms. Stick and leaf insects predominantly inhabit climatically warm regions. The climate influences the energy requirements of organisms, leading to metabolic adaptations [51]. Mitochondria play a vital role in energy production and varying climatic conditions serve as potential selective mechanisms driving the evolution of mitogenomes [108]. Ecological niche modeling has confirmed that climate was important in driving the evolution of two lowland stick and leaf insects in New Zealand (*Argosarchus horridus* and *Clitarchus hookeri*) [109]. *Ph. liaoningensis* is distributed in both southern and northern regions of China. It extends northward to Inner Mongolia and Liaoning, which have temperate continental and temperate monsoon climates. Compared to species distributed in lower latitudes, those in higher latitudes exhibit enhanced strategies for low-temperature tolerance.

The mitochondria produce the most crucial energy carrier (ATP) and encode 13 PCGs involved in the respiratory chain [48]. Positive selection has played a significant role in the evolution of mitochondrial genes. Some studies suggest that mitochondria can be positively selected as a result of physiological or environmental changes [110,111]. To investigate whether the 13 PCGs of *Ph. liaoningensis* living in high-latitude regions are subject to low temperature selection, this study conducted selection pressure analyses using *Ph. liaoningensis* collected from Anshan City, Liaoning Province, China, as the foreground branch. Two positive selection sites were detected in *Ph. liaoningensis* using the branch-site model. The 149th amino acid residue (Valine) in the *ND2* gene and the 84th residue (Leucine) in the *ND4L* gene were positively selected. The former amino acid residue diversity on the background branches may be caused by neutral evolution, meaning that these sites are not under selective pressure, but rather mutations have accumulated randomly due to genetic drift. The latter has a higher BEB value and encodes a positive charge acidic amino acid (aspartic acid) at this site on the background branch *ND4L*. Both *ND2* and *ND4L* belong to the subunits of complex I, corresponding to the hydrophobic components of complex I, which is the first complex in the respiratory chain and is responsible for transferring electrons from NADH to coenzyme Q and establishing a proton gradient [112]. It plays a pivotal role in the process of cellular energy production. *ND2* is considered to be one of the candidate genes for the proton transport mechanism and affects the assembly and stability of complex I [113]. *ND4L* constitutes the membrane domain of complex I and has some transmembrane helices, that are involved in the regulation and mediation of electron transport [114]. In *Ph. liaoningensis*, positive selection sites on *ND2* and *ND4L* suggested that adaptive changes in amino acids may affect the function of proteins under low-temperature stress, thus affecting the efficiency of proton gradient formation [115]. Xu et al. explored whether the mitochondrial gene of Heptageniidae from Ottawa, Canada, was selected by a low-temperature environment and found that there were 27 positive selection sites using the branch-site model [57]. Sun et al. detected positive selection sites on the *CYTB* and *ATP6* genes in *Tetranychus truncatus* distributed in different climatic regions using the branch-site model [56]. In the present study, two sites were positively selected, and it can be speculated that mitochondrial genes have an adaptive evolution in response to low-temperature environmental changes.

## 5. Conclusions

Currently, the published mitogenome data for Phasmatodea are very limited and include species from only nine families. We sequenced five mitochondrial genomes, including the first complete mitogenomes of *Pseudophasma* and *Acanthophasma*. This enhances our understanding of Phasmatodea mitogenomes and provides support for resolving their phylogenetic relationships. Mitochondrial genes have been widely used in molecular phylogenetics and have many advantages. They are helpful for constructing robust phylogenetic trees with complete (100%) taxa on sampling. In this study, five reliable fossil calibration points were used to support the conclusion that existing stick and leaf insects originated in the Jurassic and diversified in the Paleogene. Furthermore, the selection pressure analysis for *Ph. liaoningensis* collected from a high-latitude region showed that both *ND2* and *ND4L* had one positive selection site in this species.

## Figures and Tables

**Table 1:** Detailed information on the five Phasmatodean species collected in this study.

Family	Subfamily	Species	Length	Accession No.	Collected Location
Pseudophasmatidae	Pseudophasmatinae	*Pseudophasma subapterum* Redtenbacher, 1906	15,746 bp	PP769804	Tachira, Venezuela
Lonchodidae	Lonchodinae	*Phraortes liaoningensis* Chen & He, 1991	16,744 bp	PP769803	Liaoning, China
	Necrosciinae	*Sipyloidea biplagiata* Redtenbacher, 1908	16,103 bp	PP769805	Guangxi, China
		*Micadina brevioperculina* Bi, 1992	16,747 bp	PP769802	Guangxi, China
		*Acanthophasma brevicercum* Ho, 2020	16,476 bp	PP769801	Yunnan, China

**Table 2:** Fossil calibrations of the divergence time estimate.

Fossil	Minimum Age (mya)	Soft Maximum Age (mya)	Calibration Node	Reference
*Adjacivena rasnitsyni*	162.50	295.00	Stem Phasmatodea	[76]
*Tumefactipes prolongates*	98.17	241.50	Stem Timematidae	[77]
*Eophasmodes oregonense*	43.47	130.80	Crown Pseudophasmatidae	[37]
*Eophyllium messelense*	47.00	66.40	Stem phylliinae	[78]
*Araripephasma reliquum*	115.00	122.46	Crown Euphasmatodea	[52]

**Table 3:** Positive selection analysis of 13 mitochondrial PCGs based on the branch-site model. “*” indicates that the BEB value is greater than 0.95, and “**” indicates that the BEB value is greater than 0.99.

Model	Ln L	Estimates of Parameters					Model Compared	LRT *p*-Value	Positive Sites
Model A	−271,988.545985	Site class	0	1	2a	2b	Model A vs. Model A_null_	0.014784065	2483 D 0.996 **
		f	0.92110	0.07824	0.00061	0.00005			
		*ω*0	0.05097	1.00000	0.05097	1.00000			2107 A 0.967 *
		*ω*1	0.05097	1.00000	61.44810	61.44810			
Model A_null_	−271,991.516995	/							

## Data Availability

Data to support this study are available from the National Center for Biotechnology Information (https://www.ncbi.nlm.nih.gov) (accessed on 20 May 2024). The GenBank numbers are PP769801–PP769805.

## Supplementary Materials

The following supporting information can be downloaded at: https://www.mdpi.com/article/10.3390/insects15110858/s1, Figure S1: Relative synonymous codon usage (RSCU) of the five sequenced mitogenomes in this study; Table S1: Specific primers used to amplify the mitogenomes of *Ph. Liaoningensis*; Table S2: Information on 55 stick and leaf insects and two outgroup species used for phylogenetic analyses; Table S3: Best partitioning scheme and optimal substitution model of nt123 dataset; Table S4: Best partitioning scheme and optimal substitution model of nt123_rRNA dataset; Table S5: Location of features in five mitogenomes in this study; Table S6: Base composition of mitogenomes of the five species sequenced in this study; Table S7: Base composition of 13 mitochondrial PCGs of the five species sequenced in this study; Table S8: Amino acids use frequencies of proteins encoded by newly sequenced mitogenomes; Table S9: Codon saturation analysis of 13PCGs; Table S10: Selection pressure analysis of mitochondrial PCGs based on the branch model.
