# Supplementary material for: Insight into the Phylogenetic Relationships of Phasmatodea and Selection Pressure Analysis of Phraortes liaoningensis Chen & He, 1991 (Phasmatodea: Lonchodidae) Using Mitogenomes
Source: Insects. 2024 Nov 3;15(11):858. doi: 10.3390/insects15110858 (PMC11595267; doi:10.3390/insects15110858)
Supplement: Supplementary file 1 [file insects-15-00858-s001.zip › TableS1.pdf]

Table S1. Specific primers used to amplify the mitogenomes of *Phraortes liaoninggensis*.

Number	Primer name	Sequence (5’-3’)	Product length (bp)
1	14ZJC1-J-975	CAAGCAAGACTAAACTTAAAGGA	1251
	14ZJC1-N-2226	TGGGCTCATACTACAAAACC	
2	14ZJC1-J-3206	TATGCTACACCATCACTAC	4268
	14ZJC1-N-7474	CTAATCGTGTTGGTGACTGT	
3	14ZJC1-J-8034	TAAGGGAAATCAATGCAA	653
	14ZJC1-N-8687	TGAGGCTCCTGTATCTGG	
4	14ZJC1-J-10418	GTCGGAATGTAAATAATGGGTG	548
	14ZJC1-N-10966	GGCTGGGGTAAAGTTATCTGG	
5	14ZJCI-J-13546	CTGATACAAAAGGTACGAAA	546
	14ZJC1-N-14092	AGTCCTCGTTTTATCTTTCT	
6	14ZJC1-J-13871	GTGACGGGCGATATGTAC	3575
	14ZJC1-N-704	TCGTAATGATGTTTGGTTT	
