# Supplementary material for: Insight into the Phylogenetic Relationships of Phasmatodea and Selection Pressure Analysis of Phraortes liaoningensis Chen & He, 1991 (Phasmatodea: Lonchodidae) Using Mitogenomes
Source: Insects. 2024 Nov 3;15(11):858. doi: 10.3390/insects15110858 (PMC11595267; doi:10.3390/insects15110858)
Supplement: Supplementary file 1 [file insects-15-00858-s001.zip › TableS10.pdf]

Table S10. Selection pressure analysis of mitochondrial protein-coding genes based on the branch model.

Model	Ln L	Estimates of parameters		Model compared	LRT P-value	Omega for Foreground Branch
Two ratio Model 2	-275074.482939	ω0= 0.04992	ω1= 0.02157	Model 0 vs. Two ratio Model 2	0.000000000	ω1=0.02157
Model 0	-275094.773373	ω= 0.04958				
