# Supplementary material for: Insight into the Phylogenetic Relationships of Phasmatodea and Selection Pressure Analysis of Phraortes liaoningensis Chen & He, 1991 (Phasmatodea: Lonchodidae) Using Mitogenomes
Source: Insects. 2024 Nov 3;15(11):858. doi: 10.3390/insects15110858 (PMC11595267; doi:10.3390/insects15110858)
Supplement: Supplementary file 1 [file insects-15-00858-s001.zip › TableS2.pdf]

Table S2. Information on 55 stick and leaf insects and two outgroup species used for phylogenetic analysis.

Family	Subfamily	Species	Accession no.	Reference
Aschiphasmatidae	Aschiphasmatinae	*Nanhuaphasma hamicercum*	MZ312646	[64]
		Orthomeria smaragdinum	MZ31264	[64]
Bacillidae	Bacillinae	*Bacillus rossius*	GU001956	[19]
		Bacillus atticus	GU001955	[19]
Heteropterygidae	Dataminae	*Orestes mouhotii*	AB477462	[66]
		Orestes guangxiensis	MW450873	[22]
	Obriminae	*Aretaon asperrimus*	SRR1172270	[14]
		Sungaya inexpectata	OQ682523	[24]
	Heteropteryginae	*Heteropteryx dilatata*	AB477468	[66]
Lonchodidae	Lonchodinae	*Phraortes illepidus*	AB477460	[66]
		Phraortes lii	ON493672	Directly Submitted
		Phraortes lianzhouensis	OQ682528	[24]
		Phraortes sp. Miyako Island	AB477465	[66]
		Phraortes sp. Iriomote Island	AB477464	[66]
		Stheneboea repudiosa	OQ682531	[24]
		Megalophasma granulatum	KY124331	[67]
		Eurycantha calcarata	MW915467	[68]
		Carausius sp.	OQ682524	[24]
		Carasisus morosus	SRR3211828	[14]
	Necrosciinae	*Micadina phluctainoides*	AB477466	[66]
		Marmessoidea bispina	OQ682527	[24]
		Sipyloidea sipylus	AB477470	[66]
		Calvisia medogensis	KY124330	[67]
		Lopaphus albopunctatus	OQ682525	[24]
		Lopaphus sphalerus	OQ682526	[24]
		Sosibia gibba	OM257176	[65]
		Sosibia ovata	OM257177	[65]

		Neohirasea japonica	AB477469	[66]
		Neohirasea stephanus	OL405132	Directly Submitted
Phasmatidae	Megacraniinae	*Megacrania alpheus adan*	AB477471	[66]
	Extatosomatinae	*Extatosoma tiaratum*	AB642680	[66]
	Phasmatinae	*Acanthoxyla* sp.	SRR2089887	[14]
		Clitarchus hookeri	SRR3080266	[14]
	Clitumninae	*Entoria okinawaensis*	AB477459	[66]
		Phobaeticus serratipes	AB477467	[66]
		Ramulus hainanense	FJ156750	Directly Submitted
		Ramulus irregulariterdentatus	AB477463	[66]
		Rumulus artemis	SRR1172422	[14]
		Medauroidea extradentata	SRR1172394	[14]
		Phryganistria guangxiensis	MW450875	[22]
	Pachymorphinae	*Niveaphasma anulata*	SRR2089878	[14]
		Tectarchus ovobsessus	SRR2089893	[14]
		Tectarchus salebrosus	SRR2089908	[14]
		Micrarchus hystriculeus	SRR1054191	[14]
		Micrarchus sp	SRR1054193	[14]
Phylliidae	Phylliinae	*Cryptophyllium tibetense*	KX091862	Directly Submitted
		Cryptophyllium tibetense	KY124332	Directly Submitted
		Cryptophyllium westwoodii	MW229063	Directly Submitted
		Pulchriphyllium bioculatum	OQ682529	[24]
		Pulchriphyllium giganteum	AB477461	[66]
		Pulchriphyllium giganteum	OQ682530	[24]
		Phyllium siccifollium	ERR392012	[14]
Pseudophasmatidae	Pseudophasmatinae	*Peruphasma schultei*	MW450874	[22]
		Peruphasma schultei	SRR1002984	[14]
Timematidae	Timematinae	*Timema californicum*	DQ241799	[63]
Grylloblattidae		*Grylloblatta sculleni*	DQ241796	[63]

Mantophasmatidae	*Sclerophasma paresisense*	DQ241798	[63]
