# Supplementary material for: Insight into the Phylogenetic Relationships of Phasmatodea and Selection Pressure Analysis of Phraortes liaoningensis Chen & He, 1991 (Phasmatodea: Lonchodidae) Using Mitogenomes
Source: Insects. 2024 Nov 3;15(11):858. doi: 10.3390/insects15110858 (PMC11595267; doi:10.3390/insects15110858)
Supplement: Supplementary file 1 [file insects-15-00858-s001.zip › TableS4.pdf]

Table S4. Best partitioning scheme and optimal substitution model of nt123_rRNA dataset.

Subset	Subset Partitions	Best Model
Partition 1	16SrRNA	GTR+I+G
Partition 2	12SrRNA, ND1_codon1, ND5_codon1, ND4_codon1, ND4L_codon1	GTR+I+G
Partition 3	ND6_codon1, ATP8_codon2, ND3_codon1, ATP6_codon1, ND2_codon1, ATP8_codon1	GTR+I+G
Partition 4	Cytb_codon2, COX3_codon2, COX2_codon2, ND6_codon2, ND3_codon2, ND2_codon2, ATP6_codon2	GTR+I+G
Partition 5	ATP6_codon3, ND3_codon3, ATP8_codon3, ND6_codon3, Cytb_codon3	GTR+I+G
Partition 6	COX1_codon1	GTR+I+G
Partition 7	COX1_codon2	TVM+I+G
Partition 8	COX1_codon3, ND2_codon3	GTR+I+G
Partition 9	Cytb_codon1, COX3_codon1, COX2_codon1	GTR+I+G
Partition 10	COX2_codon3, COX3_codon3	HKY+I+G
Partition 11	ND4L_codon2, ND5_codon2, ND4_codon2, ND1_codon2	GTR+I+G
Partition 12	ND4L_codon3, ND4_codon3, ND1_codon3, ND5_codon3	GTR+I+G
