# Supplementary material for: Insight into the Phylogenetic Relationships of Phasmatodea and Selection Pressure Analysis of Phraortes liaoningensis Chen & He, 1991 (Phasmatodea: Lonchodidae) Using Mitogenomes
Source: Insects. 2024 Nov 3;15(11):858. doi: 10.3390/insects15110858 (PMC11595267; doi:10.3390/insects15110858)
Supplement: Supplementary file 1 [file insects-15-00858-s001.zip › TableS5.pdf]

Table S5. Location of features in newly sequenced mitogenomes.

A. *Sipyloidea biplagiat*

Gene	Strand	Position	Length (nuc.)	Anticodon	Start codon	Stop codon	Intergenic nucleotides
$tRNA^{Ile}$	+	1-68	68	ATC			-3
$tRNA^{Gln}$	-	66-134	69	CAA			+2
$tRNA^{Met}$	+	137-203	67	ATG			0
ND2	+	204-1202	999		ATT	TAA	-2
$tRNA^{Trp}$	+	1201-1266	66	TGA			-8
$tRNA^{Cys}$	-	1259-1322	64	TGC			0
$tRNA^{Tyr}$	-	1323-1389	67	TAC			+1
COX1	+	1391-2924	1534		ATG	T	0
$tRNA^{Leu2(UUA)}$	+	2925-2988	64	TTA			0
COX2	+	2989-3658	670		ATA	T	0
$tRNA^{Lys}$	+	3659-3728	70	AAG			-1
$tRNA^{Asp}$	+	3728-3796	65	GAC			0
ATP8	+	3797-3955	159		ATT	TAA	-4
ATP6	+	3952-4623	672		ATA	TAA	-1
COX3	+	4623-5408	786		ATG	TAA	-1
$tRNA^{Gly}$	+	5408-5470	63	GGA			0
ND3	+	5471-5822	352		ATT	T	0
$tRNA^{Ala}$	+	5823-5885	63	GCA			0
$tRNA^{Arg}$	+	5886-5949	64	CGA			+2
$tRNA^{Asn}$	+	5952-6017	66	AAC			0
$tRNA^{Ser1}$	+	6018-6085	68	AGC			0
$tRNA^{Glu}$	+	6086-6150	65	GAA			+2
$tRNA^{Phe}$	-	6153-6216	64	TTC			-1
ND5	-	6216-7940	1725		ATC	TAA	0
$tRNA^{His}$	-	7941-8004	64	CAC			-1
ND4	-	8004-9335	1332		ATG	TAA	-7
ND4L	-	9329-9616	288		ATA	TAA	+10
$tRNA^{Thr}$	+	9627-9690	64	ACA			0
$tRNA^{Pro}$	-	9691-9755	65	CCA			+2
ND6	+	9758-10237	480		ATT	TAA	-1
Cytb	+	10237-11370	1134		ATG	TAA	-2
$tRNA^{Ser2}$	+	11369-11436	68	TCA			-2
ND1	-	11435-12400	966		ATA	TAA	+3
$tRNA^{Leu1(CUA)}$	-	12404-12469	66	CTA			0
16S rRNA	-	12470-13750	1281				0
$tRNA^{Val}$	-	13751-13819	69	GTA			0
12S rRNA	-	13820-14596	777				0
CR		14597-16103	1507				

B. *Micadina brevioperculina*

Gene	Strand	Position	Length (nuc.)	Anticodon	Start codon	Stop codon	Intergenic nucleotides
$tRNA^{Ile}$	+	1-68	68	ATC			+3
$tRNA^{Gln}$	-	72-140	69	CAA			-1
$tRNA^{Met}$	+	140-206	67	ATG			0
ND2	+	207-1226	1020		ATT	TAA	-2
$tRNA^{Trp}$	+	1225-1291	67	TGA			-8
$tRNA^{Cys}$	-	1284-1348	65	TGC			+12
$tRNA^{Tyr}$	-	1361-1427	67	TAC			+1
COX1	+	1429-2962	1534		ATG	T	0
$tRNA^{Leu2(UUA)}$	+	2963-3027	65	TTA			0
COX2	+	3028-3697	670		ATA	T	0
$tRNA^{Lys}$	+	3698-3767	70	AAG			-1
$tRNA^{Asp}$	+	3767-3835	69	GAC			0
ATP8	+	3836-3997	162		ATC	TAA	-4
ATP6	+	3994-4668	675		ATA	TAA	-1
COX3	+	4668-5456	789		ATG	TAA	-1
$tRNA^{Gly}$	+	5456-5523	68	GGA			0
ND3	+	5524-5875	352		ATT	T	0
$tRNA^{Ala}$	+	5876-5940	65	GCA			0
$tRNA^{Arg}$	+	5941-6005	65	CGA			+1
$tRNA^{Asn}$	+	6007-6072	66	AAC			0
$tRNA^{Ser1}$	+	6073-6140	68	AGC			+1
$tRNA^{Glu}$	+	6142-6207	66	GAA			-2
$tRNA^{Phe}$	-	6206-6270	65	TTC			0
ND5	-	6271-7993	1723		ATT	T	0
$tRNA^{His}$	-	7994-8057	64	CAC			0
ND4	-	8058-9389	1332		ATG	TAA	-7
ND4L	-	9383-9673	291		GTG	TAA	+2
$tRNA^{Thr}$	+	9676-9739	64	ACA			0
$tRNA^{Pro}$	-	9740-9806	67	CCA			+1
ND6	+	9808-10281	474		ATA	TAA	+3
Cytb	+	10285-11418	1134		ATG	TAA	+6
$tRNA^{Ser2}$	+	11425-11493	69	TCA			-2
ND1	-	11492-12457	966		ATA	TAA	+3
$tRNA^{Leu1(CUA)}$	-	12461-12527	67	CTA			0
16S rRNA	-	12528-13817	1290				0
$tRNA^{Val}$	-	13818-13886	69	GTA			0
12S rRNA	-	13887-14657	771				0
CR		14658-16747	2090				

C. *Acanthophasma brevicercum*

Gene	Strand	Position	Length (nuc.)	Anticodon	Start codon	Stop codon	Intergenic nucleotides
$tRNA^{Ile}$	+	1-67	67	ATC			+3
$tRNA^{Gln}$	-	65-133	69	CAA			-1
$tRNA^{Met}$	+	133-200	68	ATG			0
ND2	+	201-1220	1020		ATT	TAA	-2
$tRNA^{Trp}$	+	1219-1285	67	TGA			-8
$tRNA^{Cys}$	-	1278-1345	68	TGC			+4
$tRNA^{Tyr}$	-	1350-1414	65	TAC			+1
COX1	+	1416-2949	1534		ATG	T	0
$tRNA^{Leu2(UUA)}$	+	2950-3014	65	TTA			0
COX2	+	3015-3681	667		ATA	T	0
$tRNA^{Lys}$	+	3682-3752	71	AAG			-1
$tRNA^{Asp}$	+	3752-3819	68	GAC			0
ATP8	+	3820-3978	159		ATT	TAA	-4
ATP6	+	3975-4649	675		ATA	TAA	-1
COX3	+	4649-5437	789		ATG	TAA	0
$tRNA^{Gly}$	+	5438-5503	66	GGA			0
ND3	+	5504-5855	352		ATT	T	0
$tRNA^{Ala}$	+	5856-5919	64	GCA			-1
$tRNA^{Arg}$	+	5919-5983	65	CGA			+2
$tRNA^{Asn}$	+	5986-6050	65	AAC			0
$tRNA^{Ser1}$	+	6051-6118	68	AGC			+1
$tRNA^{Glu}$	+	6120-6183	64	GAA			0
$tRNA^{Phe}$	-	6184-6249	66	TTC			0
ND5	-	6250-7969	1720		ATT	T	0
$tRNA^{His}$	-	7970-8033	64	CAC			0
ND4	-	8034-9365	1332		ATG	TAA	-7
ND4L	-	9359-9646	288		ATA	TAA	+5
$tRNA^{Thr}$	+	9652-9715	64	ACA			0
$tRNA^{Pro}$	-	9716-9783	68	CCA			+1
ND6	+	9785-10267	483		ATT	TAA	-1
Cytb	+	10267-11400	1134		ATG	TAA	+3
$tRNA^{Ser2}$	+	11404-11469	66	TCA			-1
ND1	-	11469-12437	969		TTG	TAA	0
$tRNA^{Leu1(CUA)}$	-	12438-12506	69	CTA			0
16S rRNA	-	12507-13801	1295				0
$tRNA^{Val}$	-	13802-13870	69	GTA			0
12S rRNA	-	13877-14631	755				0
CR		14632-16476	1845				

D. *Pseudophasma subapterum*

Gene	Strand	Position	Length (nuc.)	Anticodon	Start codon	Stop codon	Intergenic nucleotides
$tRNA^{Ile}$	+	1-67	67	ATC			+3
$tRNA^{Gln}$	-	65-133	69	CAA			0
$tRNA^{Met}$	+	134-202	69	ATG			0
ND2	+	203-1222	1020		ATT	TAA	-2
$tRNA^{Trp}$	+	1221-1285	65	TGA			-8
$tRNA^{Cys}$	-	1278-1342	65	TGC			0
$tRNA^{Tyr}$	-	1343-1408	66	TAC			+1
COX1	+	1410-2943	1534		TTG	T	0
$tRNA^{Leu2(UUA)}$	+	2944-3008	65	TTA			0
COX2	+	3009-3678	670		ATC	T	0
$tRNA^{Lys}$	+	3679-3748	70	AAG			-1
$tRNA^{Asp}$	+	3748-3812	65	GAC			0
ATP8	+	3813-3971	159		ATT	TAA	-4
ATP6	+	3968-4642	675		ATA	TAA	-1
COX3	+	4642-5430	789		ATG	TAA	-1
$tRNA^{Gly}$	+	5430-5492	63	GGA			0
ND3	+	5493-5844	352		ATA	T	0
$tRNA^{Ala}$	+	5845-5908	64	GCA			0
$tRNA^{Arg}$	+	5909-5973	65	CGA			+2
$tRNA^{Asn}$	+	5976-6040	65	AAC			0
$tRNA^{Ser1}$	+	6041-6108	68	AGC			0
$tRNA^{Glu}$	+	6109-6174	66	GAA			-2
$tRNA^{Phe}$	-	6173-6236	64	TTC			0
ND5	-	6237-7959	1723		ATT	T	0
$tRNA^{His}$	-	7960-8022	63	CAC			-1
ND4	-	8022-9353	1332		ATG	TAA	-7
ND4L	-	9347-9634	288		ATA	TAA	+5
$tRNA^{Thr}$	+	9640-9702	63	ACA			0
$tRNA^{Pro}$	-	9703-9765	63	CCA			+1
ND6	+	9767-10240	474		ATT	TAA	-1
Cytb	+	10240-11379	1140		ATG	TAG	-2
$tRNA^{Ser2}$	+	11378-11443	66	TCA			+1
ND1	-	11445-12408	964		TTG	T	0
$tRNA^{Leu1(CUA)}$	-	12409-12475	67	CTA			0
16S rRNA	-	12476-13754	1279				0
$tRNA^{Val}$	-	13755-13823	69	GTA			0
12S rRNA	-	13824-14596	773				0
CR		14597-15746	1150				

E. *Phraortes liaoninggensis*

Gene	Strand	Position	Length (nuc.)	Anticodon	Start codon	Stop codon	Intergenic nucleotides
$tRNA^{Ile}$	+	1-66	66	ATC			+2
$tRNA^{Gln}$	-	69-137	69	CAA			-1
$tRNA^{Met}$	+	137-200	64	ATG			0
ND2	+	201-1199	999		ATC	TAA	-2
$tRNA^{Trp}$	+	1198-1263	66	TGA			-8
$tRNA^{Cys}$	-	1256-1317	62	TGC			0
$tRNA^{Tyr}$	-	1318-1380	63	TAC			+1
COX1	+	1382-2915	1534		ATG	T	0
$tRNA^{Leu2(UUA)}$	+	2916-2980	65	TTA			0
COX2	+	2981-3647	667		ATA	T	0
$tRNA^{Lys}$	+	3648-3716	69	AAG			0
$tRNA^{Asp}$	+	3717-3781	65	GAC			+1
ATP8	+	3783-3941	159		ATG	TAA	-4
ATP6	+	3938-4612	678		ATA	TAA	-1
COX3	+	4612-5398	787		ATG	T	0
$tRNA^{Gly}$	+	5399-5461	63	GGA			+3
ND3	+	5465-5815	351		ATA	TAG	-2
$tRNA^{Ala}$	+	5814-5876	63	GCA			0
$tRNA^{Arg}$	+	5877-5942	66	CGA			+3
$tRNA^{Asn}$	+	5946-6011	66	AAC			0
$tRNA^{Ser1}$	+	6012-6078	67	AGC			0
$tRNA^{Glu}$	+	6079-6143	65	GAA			-2
$tRNA^{Phe}$	-	6142-6205	64	TTC			+1
ND5	-	6207-7929	1723		ATT	T	0
$tRNA^{His}$	-	7930-7993	64	CAC			-1
ND4	-	7993-9318	1326		ATG	TAG	-7
ND4L	-	9328-9600	273		TTG	TAA	+9
$tRNA^{Thr}$	+	9605-9667	63	ACA			0
$tRNA^{Pro}$	-	9668-9731	64	CCA			+1
ND6	+	9733-10203	471		ATA	TAA	-1
Cytb	+	10203-11333	1131		ATG	TAG	-2
$tRNA^{Ser2}$	+	11332-11399	68	TCA			+1
ND1	-	11401-12367	967		TTG	T	0
$tRNA^{Leu1(CUA)}$	-	12368-12437	70	CTA			0
16S rRNA	-	12438-13717	1280				0
$tRNA^{Val}$	-	13718-13786	69	GTA			0
12S rRNA	-	13787-14532	746				0
CR		14533-16744	2212				
