# Supplementary material for: Insight into the Phylogenetic Relationships of Phasmatodea and Selection Pressure Analysis of Phraortes liaoningensis Chen & He, 1991 (Phasmatodea: Lonchodidae) Using Mitogenomes
Source: Insects. 2024 Nov 3;15(11):858. doi: 10.3390/insects15110858 (PMC11595267; doi:10.3390/insects15110858)
Supplement: Supplementary file 1 [file insects-15-00858-s001.zip › TableS6.pdf]

Table S6. Base composition of mitogenomes of the five species sequenced in this study.

Family	Species	Region	Whole genome	PCGs		tRNA		rRNA	Control Region
		Strand	+	+	-	+	-	-	+
Lonchodidae	*Sipyloidea biplagiata*	Length(bp)	16,103	6783	4311	925	528	2058	1507
		A+T%	77.3	75.1	78.3	81.4	78.4	79.5	78.7
		AT-Skew	0.174	0.081	-0.352	0.078	-0.092	-0.223	0.080
		GC-Skew	-0.200	-0.167	0.268	0.023	0.421	0.291	-0.099
Pseudophasmatidae	*Pseudophasma subapterum*	Length(bp)	15,746	6813	4305	921	526	2052	1150
		A+T%	74.1	70.5	75.1	77.5	76.2	77.6	82.1
		AT-Skew	0.195	0.073	-0.393	0.084	-0.092	-0.257	0.164
		GC-Skew	-0.211	-0.149	0.279	-0.014	0.360	0.297	-0.367
Lonchodidae	*Phraortes liaoninggensis*	Length(bp)	16,744	6774	4305	912	529	2026	2212
		A+T%	77.4	75.3	78.4	79.2	78.0	78.1	80.1
		AT-Skew	0.171	0.080	-0.368	0.080	-0.085	-0.219	0.076
		GC-Skew	-0.156	-0.129	0.235	-0.011	0.276	0.278	0.000
Lonchodidae	*Micadina brevioperculina*	Length(bp)	16,747	6807	4311	937	533	2061	2090
		A+T%	77.6	75.3	78.3	79.4	78.3	79.7	79.9
		AT-Skew	0.165	0.062	-0.353	0.078	-0.103	-0.199	0.126
		GC-Skew	-0.139	-0.118	0.262	0.036	0.345	0.311	0.204
Lonchodidae	*Acanthophasma brevicercum*	Length(bp)	16,476	6810	4308	928	538	2050	1845
		A+T%	78.2	76.3	78.9	79.2	78.5	80	80.7
		AT-Skew	0.200	0.108	-0.374	0.078	-0.118	-0.228	0.175
		GC-Skew	-0.138	-0.130	0.253	0.026	0.362	0.278	0.337
