# Supplementary material for: Insight into the Phylogenetic Relationships of Phasmatodea and Selection Pressure Analysis of Phraortes liaoningensis Chen & He, 1991 (Phasmatodea: Lonchodidae) Using Mitogenomes
Source: Insects. 2024 Nov 3;15(11):858. doi: 10.3390/insects15110858 (PMC11595267; doi:10.3390/insects15110858)
Supplement: Supplementary file 1 [file insects-15-00858-s001.zip › TableS7.pdf]

Table S7. Base composition of 13 mitochondrial protein-coding genes of the five species sequenced in this study.

Species	Gene	ATP6	ATP8	COX1	COX2	COX3	Cytb	ND1	ND2	ND3	ND4	ND4L	ND5	ND6
	Strand	+	+	+	+	+	+	-	+	+	-	-	-	+
Sipyloidea biplagiata	length	672	159	1534	670	786	1134	966	999	352	1332	288	1725	480
	A+T%	76.3	85.6	70.8	73.3	72.4	73.4	77.2	80.7	78.4	79	81.2	77.8	81.3
	AT-Skew	0.072	0.206	0.026	0.149	0.033	0.041	-0.408	0.138	0.08	-0.359	-0.402	-0.307	0.154
	GC-Skew	-0.208	-0.217	-0.058	-0.162	-0.097	-0.179	0.255	-0.281	-0.289	0.243	0.407	0.274	-0.422
Micadina brevioperculina	length	675	162	1534	670	789	1134	966	1020	352	1332	291	1723	474
	A+T%	76.2	84.6	70.6	72.5	72.9	72.9	78.4	82.1	77.2	78.7	83.1	77.1	83.5
	AT-Skew	0.066	0.255	-0.017	0.107	-0.026	0.034	-0.367	0.128	0.088	-0.361	-0.388	-0.333	0.172
	GC-Skew	-0.18	-0.44	-0.027	-0.141	-0.056	-0.13	0.288	-0.18	-0.25	0.232	0.306	0.264	-0.205
Phraortes liaoninggensis	length	678	159	1534	667	787	1131	967	999	351	1326	291	1723	471
	A+T%	76.2	86.2	69.7	73.6	72.4	73.8	77.5	81.9	76.9	78.3	81.1	78.5	84
	AT-Skew	0.087	0.241	-0.004	0.141	0.028	0.037	-0.413	0.15	0.111	-0.369	-0.373	-0.343	0.162
	GC-Skew	-0.217	-0.273	-0.043	-0.136	-0.069	-0.115	0.257	-0.144	-0.259	0.213	0.345	0.224	-0.467
Acanthophasma brevicercum	length	675	159	1534	667	789	1134	969	1020	352	1332	288	1720	483
	A+T%	77.7	88.1	70.9	75.2	74.2	73.6	77.8	82.5	78.4	78.4	82.6	79.3	84.3
	AT-Skew	0.092	0.229	0.051	0.159	0.056	0.085	-0.43	0.171	0.087	-0.395	-0.378	-0.326	0.174
	GC-Skew	-0.258	-0.368	-0.031	-0.139	-0.059	-0.144	0.265	-0.258	-0.263	0.254	0.32	0.238	-0.395
Pseudophasma subapterum	length	678	159	1534	670	789	1140	964	1020	352	1332	288	1723	474
	A+T%	70	84.2	66.2	71.3	67.2	68.2	73.8	75.1	72.7	75.5	79.5	74.7	78
	AT-Skew	0.095	0.149	-0.003	0.088	0.057	0.05	-0.423	0.146	0.086	-0.414	-0.424	-0.357	0.103
	GC-Skew	-0.261	-0.12	-0.071	-0.104	-0.058	-0.152	0.273	-0.197	-0.271	0.276	0.322	0.28	-0.404
