# Supplementary material for: Insight into the Phylogenetic Relationships of Phasmatodea and Selection Pressure Analysis of Phraortes liaoningensis Chen & He, 1991 (Phasmatodea: Lonchodidae) Using Mitogenomes
Source: Insects. 2024 Nov 3;15(11):858. doi: 10.3390/insects15110858 (PMC11595267; doi:10.3390/insects15110858)
Supplement: Supplementary file 1 [file insects-15-00858-s001.zip › TableS8.pdf]

Table S8. Amino acids use frequencies of proteins encoded by newly sequenced mitogenomes.

species	*Sipyloidea biplagiata*		*Micadina brevioperculina*		*Acanthophasma brevicercum*		*Phraortes liaoninggensis*		*Pseudophasma subapterum*	
AA	Count	%	Count	%	Count	%	Count	%	Count	%
Phe(F)	336	9.11	331	8.95	353	9.55	334	9.06	344	9.3
Leu2(L2)	386	10.47	415	11.23	411	11.12	383	10.39	368	9.95
Leu1(L1)	113	3.06	87	2.35	73	1.97	111	3.01	136	3.68
Ile(I)	376	10.2	373	10.09	369	9.98	366	9.93	345	9.33
Met(M)	359	9.73	361	9.76	390	10.55	369	10.01	312	8.44
Val(V)	173	4.69	173	4.68	155	4.19	182	4.94	204	5.52
Ser2(S2)	224	6.07	220	5.95	226	6.11	227	6.16	229	6.19
Pro(P)	130	3.52	133	3.6	128	3.46	134	3.64	133	3.6
Thr(T)	210	5.69	207	5.6	206	5.57	198	5.37	218	5.9
Ala(A)	110	2.98	124	3.35	112	3.03	113	3.07	135	3.65
Tyr(Y)	179	4.85	182	4.92	177	4.79	182	4.94	175	4.73
His(H)	64	1.74	67	1.81	64	1.73	68	1.85	63	1.7
Gln(Q)	66	1.79	64	1.73	63	1.7	62	1.68	62	1.68
Asn(N)	190	5.15	192	5.19	208	5.63	194	5.26	193	5.22
Lys(K)	110	2.98	110	2.98	113	3.06	111	3.01	106	2.87
Asp(D)	74	2.01	72	1.95	71	1.92	75	2.04	76	2.06
Glu(E)	76	2.06	75	2.03	76	2.06	76	2.06	78	2.11
Cys(C)	43	1.17	45	1.22	46	1.24	51	1.38	49	1.33
Trp(W)	92	2.49	96	2.6	89	2.41	90	2.44	98	2.65
Arg(R)	54	1.46	52	1.41	51	1.38	52	1.41	52	1.41
Ser1(S1)	121	3.28	115	3.11	120	3.25	107	2.9	119	3.22
Gly(G)	202	5.48	203	5.49	196	5.3	200	5.43	203	5.49
