# Supplementary material for: Insight into the Phylogenetic Relationships of Phasmatodea and Selection Pressure Analysis of Phraortes liaoningensis Chen & He, 1991 (Phasmatodea: Lonchodidae) Using Mitogenomes
Source: Insects. 2024 Nov 3;15(11):858. doi: 10.3390/insects15110858 (PMC11595267; doi:10.3390/insects15110858)
Supplement: Supplementary file 1 [file insects-15-00858-s001.zip › TableS9.pdf]

Table S9. Codon saturation analysis of 13PCGs.

codon	NumOTU	Iss	Issc	P
1st	4	0.252	0.849	0.0000
	8	0.251	0.840	0.0000
	16	0.259	0.826	0.0000
	32	0.262	0.808	0.0000
2nd	4	0.140	0.849	0.0000
	8	0.139	0.840	0.0000
	16	0.135	0.826	0.0000
	32	0.145	0.808	0.0000
3rd	4	0.602	0.849	0.0000
	8	0.605	0.840	0.0000
	16	0.610	0.826	0.0000
	32	0.611	0.808	0.0000

Note: Iss.c indicates critical index of substitution saturation; Iss indicates index of substitution saturation; P indicates significance level.
